# A positive feedback between IDO1 metabolite and COL12A1 via MAPK pathway to promote gastric cancer metastasis

**DOI:** 10.1186/s13046-019-1318-5

**Published:** 2019-07-17

**Authors:** Zhen Xiang, Jun Li, Shuzheng Song, Jiexuan Wang, Wei Cai, Wenjun Hu, Jun Ji, Zhenggang Zhu, Lu Zang, Ranlin Yan, Yingyan Yu

**Affiliations:** Department of Surgery of Ruijin Hospital, and Shanghai Key Laboratory for Gastric Neoplasms, Shanghai Jiao Tong University School of Medicine, Shanghai, China

**Keywords:** Gastric cancer, WGCNA, IDO1, COL12A1, Lymph node metastasis

## Abstract

**Background:**

IDO1 (Indoleamine 2,3-dioxygenase 1) inhibits host anti-tumor immune response by exhausting tryptophan in tumor microenvironment, but the pathogenic mechanisms of IDO1 in gastric cancer (GC) cells need to be further explored.

**Methods:**

The aim of this study was to use CCLE (Cancer Cell Line Encyclopedia) transcriptomic data of GC cell lines for WGCNA (Weighted Gene Co-expression Network Analysis) analysis, and explore the potential functions and mechanisms of IDO1 in GC progression in vitro and in vivo.

**Results:**

The higher expression level of IDO1 was identified in 4 out of 7 GC cell lines. Increased IDO1 expression strongly promoted cell migration via its metabolite kynurenine and was associated with pathways of immune activation according to GSEA (Gene Set Enrichment Analysis). The functions of *IDO1* were closely associated with extracellular matrix, collagen metabolic and catabolic process by WGCNA analysis. Among five hub genes (*AXL*, *SGCE*, *COL12A1*, *ANTXR1*, *LOXL2*), *COL12A1* and *LOXL2* were upregulated in GC tissues. IDO1 disclosed positive correlation with six collagen genes by coefficient matrix diagram. Knockdown of IDO1 decreased the expression of LOXL2, COL6A1, COL6A2 and COL12A1 in GC cells in both mRNA and protein levels. Of them, knockdown of COL12A1 inhibited cell migration more apparently than knockdown of others. IDO1 and COL12A1 revealed synergistic efficacy on promoting cell migration via a positive feedback sustained by MAPK pathway. This bioprocess was mediated by IDO1 metabolite kynurenine and integrin β1. A popliteal lymph nodemetastasis model was established for verifying metastatic promotion of IDO1 and COL12A1 in GC.

**Conclusions:**

IDO1 and COL12A1 synergistically promoted GC metastasis. The novel findings suggested that both IDO1 and COL12A1 may be promising targets on anti-cancer treatment in GC.

**Electronic supplementary material:**

The online version of this article (10.1186/s13046-019-1318-5) contains supplementary material, which is available to authorized users.

## Background

Indoleamine 2,3-dioxygenase 1 (IDO1) is the rate-limiting enzyme for catalyzing tryptophan to kynurenine, and plays a key role in metabolism of tryptophan [1]. The higher level of IDO1 could result in the lack of tryptophan in tumor microenvironment [2–4], which directly reduces the proliferation ability and functions of immune cells. Therefore, increased IDO1 level could be taken as a reason of immune escape in cancers [5]. Recently, IDO1 is becoming a focus in the area of cancer therapy. Liu et al. found that IDO1 could promote dormancy of tumor-repopulating cells by activating IDO-kynurenine-AhR metabolic circuitry, a process closely related to tumor recurrence and metastasis [6]. They proposed that activation of STAT3/p53 pathway could reverse this process and induce apoptosis in dormant tumor-repopulating cells [7]. Besides, p53 alleviated invasion and migration ability of lung cancer cells by inhibiting the expression of IDO1 in cancer cells [8]. Brito et al. reported that IDO inhibitor (1-methyl-D-tryptophan) could attenuate invasion and migration capacity of bladder cancer cells by inhibiting TGFβ-induced EMT [9]. Moreover, IDO1 metabolites could promote growth of colon cancer by activating β-catenin signaling pathway [10]. Down-regulation of IDO1 could enhance gemcitabine sensitivity of lung cancer cell A549 [11]. In GC, high expression of IDO1 was closely related to poor prognosis [12, 13]. However, the underlying molecular mechanisms are still poorly known.

The TCGA database (https://portal.gdc.cancer.gov/) provides data of genome, transcriptome, methylation and prognosis from over 10000 cases of tumors. However, the data reflects mixed level of *IDO1* for cancer cells and stromal cells in tumor microenvironment [14]. The CCLE database collects the data of genome, transcriptome and methylation from over 1000 cancer cell lines [15]. It is suitable for exploring carcinogenic mechanisms in multiple cancer cells [16]. For instance, we reported expression levels of *ERBB2* and *RARA* in GC cell lines were positively correlated with the sensitivity of ERBB2 targeted therapy. We also found that lncRNAs *GIHCG* and *SPINT1-AS1* could regulate lapatinib sensitivity of cancer cells based on the CCLE analysis [17, 18].

In this study, we analyzed the transcriptomic data of GC cell lines by WGCNA analysis, and firstly noticed that IDO1 was positively associated with extracellular matrix expression. By further screening possible functions of hub genes, we confirmed that IDO1 and COL12A1 synergistically promoted GC metastasis by forming a positive feedback via MAPK pathway.

## Methods

### Data collection and analysis

Normalized transcriptomic data of 38 GC cell lines were extracted from CCLE database. As for the gene with multiple probes, the probe with maximum average value was selected for the further analysis. A total of 3000 most variable genes were selected to perform WGCNA analysis by using “WGCNA” package in R software. The mRNA expression levels of *IDO1*, *AXL*, *SGCE*, *COL12A1*, *ANTXR1* and *LOXL2* of 32 paired gastric mucosa and cancer tissues were abstracted from TCGA database.

### Cell lines, cell culture, siRNA and plasmid transfection, and lentiviral infection

One immortalized gastric epithelial cell line (GES-1) and 7 GC cell lines (SGC-7901, NCI-N87, AGS, MKN45, MGC-803, HGC-27 and Hs746T) were stored at Shanghai Institute of Digestive Surgery. All cell lines were cultured in RPMI-1640 medium supplemented with 10% FBS and maintained in a humidified atmosphere at 37 °C in 5% CO2. Lipofectamine 2000 reagent (Invitrogen, Carlsbad, California, USA) was used to perform siRNA (GeneChem, Shanghai, China) and plasmid (GeneChem, Shanghai, China) transfection according to the manufacturer’s instructions. The siRNA sequences were listed in Additional file 1: Table S1. The most effective siRNAs were used to establish Lentivirus-shRNA and verified by sequencing. To establish SGC-7901 cell lines stably expressing IDO1 shRNAs or/and COL12A1 shRNAs, Lentivirus-IDO1 shRNA and/or Lentivirus-COL12A1 shRNA were used to transfect cell lines, followed by puromycin (2 μg/ml) and blasticidin (10 μg/ml) treatment. HGC-27 cells stably expressing IDO1 or/and COL12A1 shRNA were also generated by lentiviral transduction and selected by puromycin (2 μg/ml) and blasticidin (10 μg/ml). All lentivirus also contained gene encoding Green Fluorescent Protein (GFP).

### Cell counting Kit-8 assay

After IDO1 siRNA or IDO1-expressing eukaryotic plasmid transfection, cancer cells were resuspended, and 5000 cells were placed in 96 well plates (100 μl/well). Forty eight hours later, Cell Counting Kit-8 was applied to examine proliferation ability (CK04, DOJINDO, Kumamoto, Japan). The OD value at 450 nm was measured by spectrophotometry (BioTek, Vermont, USA).

### Transwell assay

Fifty thousand cells were seeded onto upper chamber (BD Bioscience, San Jose, California, USA), and cultured in RPMI-1640 medium supplemented with 1% FBS. The lower of the chamber was filled with RPMI-1640 medium supplemented with 10% FBS. After incubation at 37 °C for 24 h, the cells were fixed and then stained with 0.1% crystal violet. Cells under the membrane were counted under the microscope in five high-power fields (400 ×).

### RNA sequencing and data analysis

RNA-seq libraries were prepared using the TruSeq RNA Sample Preparation kit (Illumina, San Diego, CA, USA) according to the manufacturer’s instructions. Gene counts were normalized to transcripts per million (TPM, Supplementary data). The genes with |log2 (fold change)| > 1 were used for GO analysis by clusterProfiler package in R software.

### Quantitative RT-PCR

The total RNA of cancer cells was extracted using TRIzol solution (Invitrogen, California, USA), and Reverse Transcription kit (TOYOBO, Osaka, Japan) was utilized to synthesize cDNA. Quantitative RT-PCR was performed as previous study [19]. The sequences of premiers were listed in Additional file 2: Table S2.

### Western blot

The total protein, cytoplasmic protein and nuclear protein were extracted separately, and Western blot was performed as previously described [17, 19]. The antibodies used in this study were presented in Additional file 3: Table S3. GAPDH was used as an internal control for total protein and cytoplasmic protein, while histone H3 was used as an internal control for nuclear protein.

### Immunohistochemical (IHC) staining

All removed subcutaneous tumors and lymph nodes were fixed by formalin and embedded in paraffin. The 4 μm thick slices were made to perform IHC staining by streptavidin-peroxidase method as previous study [20]. Semi-quantitative expression analysis of IHC was conducted according to the proportion and intensity of stained tumor cells, which was described as reported previously [19]. The antibodies used in this study were stated in Additional file 3: Table S3.

### Popliteal lymph node metastasis model

Popliteal lymph node metastasis model was established to evaluate metastasis ability of cancer cells, which was approved by the Research Ethics Committee of Shanghai Jiaotong University. The experimental mice were divided into four groups randomly: HGC-27_NC group, HGC-27_shCOL12A1 group, HGC-27_IDO1 group and HGC-27_IDO1 + shCOL12A1 group. A total of 5 × 10^6^ HGC-27 cells were injected into the foot-pad of left hind. The detailed steps were reported in the previous study [19]. Three weeks later, the mice were sacrificed, and primary tumors in foot pad and popliteal lymph nodes were removed for further analysis.

### Statistics

GSEA analysis was performed as previous study [17]. Other statistics were performed using GraphPad Prism 6.0 (Inc., La Jolla, CA, United States). Student’s t-test and χ2 test were performed to evaluate the difference between two groups. *P* value less than 0.05 was considered statistically significant.

## Results

### IDO1 apparently promotes GC migration

**Fig. 1:**
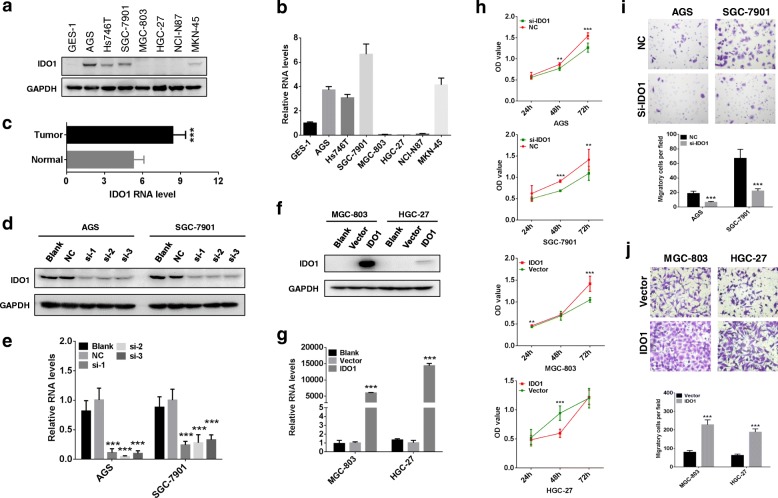
The effects of IDO1 on cell migration of GC. IDO1 expression was detected by Western blot (**a**) and qRT-PCR (**b**) in gastric epithelial cell line (GES-1) and GC cell lines (MGC-803, HGC-27, NCI-N87, AGS, Hs746T, SGC-7901 and MKN45). **c** *IDO1* RNA level in 32 paired GC and normal tissues in TCGA database (*P* < 0.001). Western blot (**d**) and qRT-PCR (**e**) were used to detect IDO1 expression after transfecting IDO1 siRNAs for 2 days in AGS and SGC-7901 cells. MGC-803 and HGC-27 were transfected by IDO1-expressing eukaryotic plasmid for 2 days, and IDO1 was examined by Western blot (**f**) and qRT-PCR (**g**). **h** Effects of IDO1 expression on GC cell proliferation of AGS, SGC-7901, MGC-803 and HGC-27. IDO1 was knocked down by siRNA transfection, and upregulated by IDO1-expressing eukaryotic plasmid transfection. **i** Knockdown of IDO1 by siRNA transfection inhibited migration of AGS and SGC-7901 (200×). **j** IDO1 up-regulation by IDO1-expressing eukaryotic plasmid transfection promoted cell migration of MGC-803 and HGC-27 (200×). “*” represented comparing with “Normal”, “NC” or “Vector” group. *P < 0.05, **P < 0.01, ***P < 0.001

To investigate the functions of IDO1 in GC cell, we firstly examined the expression of IDO1 in a gastric epithelial cell line and GC cell lines using Western blot (Fig. 1a) and qRT-PCR (Fig. 1b). The expression level of IDO1 was relatively lower in gastric epithelial cells GES-1, compared to that in GC cell lines AGS, Hs746T, SGC-7901 and MKN-45. MGC-803, HGC-27 and NCI-N87 cancer cell lines revealed lower expression level of IDO1. In TCGA database, GC tissues also showed higher mRNA level of *IDO1* than that in normal tissues (Fig. 1c, *P* < 0.001). The effects of IDO1 on cell growth and migration were examined by enforcing and decreasing IDO1 in GC cells. We successfully knocked down IDO1 in AGS and SGC-7901 cells by transient siRNA transfection (Fig. 1d and e), and enforced IDO1 expression in MGC-803 and HGC-27 with eukaryotic expressing plasmid (Fig. 1f and g). For IDO1 siRNAs, we selected the most efficient sequence (si-1) for the subsequent experiments. Cell growth (Fig. 1h, all *P* < 0.05) and migration ability of AGS and SGC-7901 cells (Fig. 1i, all *P* < 0.05) were inhibited after IDO1 siRNA transfection. Up-regulation of IDO1 promoted cell growth of MGC-803 cancer cells, but attenuated cell growth at 24 h in HGC-27 cancer cells (Fig. 1h, all P < 0.05). Nevertheless, up-regulation of IDO1 in HGC-27 and MGC-803 induced cell migration (Fig. 1j, all *P* < 0.05).

### IDO1-involved pathways in GC cell lines differ from these in GC tissues

**Fig. 2:**
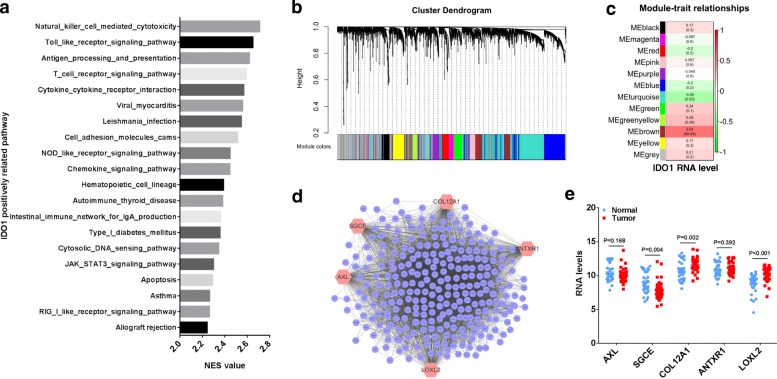
*IDO1* involved pathways based on GSEA and WGCNA analysis in GC. **a** GSEA analysis showed *IDO1*-related KEGG pathways in GC tissue of TCGA database (High expression group (*n* = 207) vs Low expression group (*n* = 208), *P* < 0.01, FDR < 0.01). **b** Identification of gene co-expression modules using transcriptomic data of GC cell lines in CCLE by average hierarchical linkage clustering; the y-axis and x-axis represented the co-expression distance and genes respectively. Modules were identified using dynamic tree cutting by dividing the dendrogram at significant branch points. Modules were displayed with different colors below the dendrogram, and gray modules indicated unassigned genes. **c** The correlation coefficients between *IDO1* mRNA level and different modules. **d** WGCNA analysis was performed using the genes in brown module. Five hub genes (*AXL*, *SGCE*, *COL12A1*, *ANTXR1* and *LOXL2*) with the degree ≥160 were identified in brown module by Cytoscape software. **e** The expression levels of *AXL*, *SGCE*, *COL12A1*, *ANTXR1* and *LOXL2* in 32 paired gastric normal and cancer tissues in TCGA database

Using TCGA RNA-seq data, we set the median value of *IDO1* mRNA level as cut-off to divide 415 cases of GC into two groups: High expression group (*n* = 207) and Low expression group (*n* = 208). The immune activation pathway of KEGG, such as T cell and NK cell were significantly enriched in High expression group by GSEA analysis (Fig. 2a, *P* < 0.01, FDR < 0.01). In order to explore the potential mechanisms of IDO1 on promoting migration of GC cell, we analyzed the transcriptomic data of 38 GC cell lines in CCLE database by WGCNA analysis. We used 3000 most variable genes to construct 12 modules (Fig. 2b). The genes enriched in these modules were listed in Additional file 4: Supplementary data. By correlation analysis, the mRNA level of *IDO1* was positively correlated with the brown module (Fig. 2c, *R* = 0.54, *P* < 0.001), in which the enriched genes were involved in the synthesis and metabolism of extracellular matrix, mainly including collagens (Supplementary data). We constructed a co-expressing network of genes in the brown module by Cytoscape software, and five hub genes of *AXL*, *SGCE*, *COL12A1*, *ANTXR1* and *LOXL2* were disclosed with the degree ≥160 (Fig. 2d). We further analyzed the expression levels of the 5 hub genes in 32 paired GC and normal tissues in TCGA database, and found that the expression levels of *LOXL2* and *COL12A1* were significantly increased in GC tissues than those in normal tissues (Fig. 2e, all *P* < 0.05).

### IDO1 positively regulates several members of collagen family

**Fig. 3:**
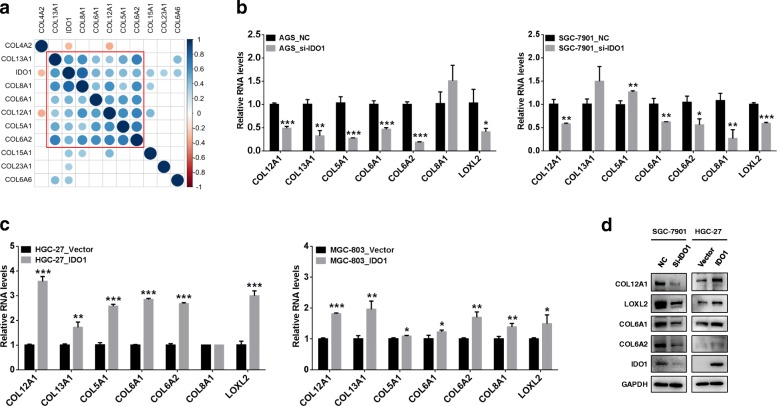
IDO1 regulates collagen genes in GC cell. **a** Correlation coefficient matrix diagram showed the correlation coefficients among IDO1 and collagen family genes included in WGCNA analysis. The genes were simultaneously clustered by hierarchical clustering order. The larger the dots, the stronger the correlation. Blank represented the *P* values ≥0.05, and other P values were less than 0.05. Effect of IDO1 down-regulation (**b**) or up-regulation (**c**) on expression of *COL12A1*, *LOXL2*, *COL6A1*, *COL6A2*, *COL5A1*, *COL8A1* and *COL13A1* in AGS and SGC-7901 or HGC-27 and MGC-803 respectively. GC cells were transfected by IDO1 siRNA or IDO1-overexpressing eukaryotic plasmid. Three days later, *COL12A1*, *LOXL2*, *COL6A1*, *COL6A2*, *COL5A1*, *COL8A1* and *COL13A1* RNA levels were detected by qRT-PCR. **d** Western blot was applied to examine IDO1, COL12A1, LOXL2, COL6A1 and COL6A2 protein levels in IDO1 shRNA stably expressing SGC-7901 and IDO1-overexpressing HGC-27 cell lines

We selected 10 collagen family genes among above 3000 most variable genes and performed WGCNA analysis. As shown in Fig. 3a, collagen gene *COL5A1* (*R* = 0.43), *COL6A1* (*R* = 0.47), *COL6A2* (*R* = 0.47), *COL8A1* (*R* = 0.79), *COL12A1* (*R* = 0.45) and *COL13A1* (*R* = 0.56) revealed positive correlation to *IDO1* (all *P* < 0.01), and well clustered. To explore the regulatory relationships between IDO1 and above genes, at the 3rd day of knockdown IDO1 in AGS and SGC-7901 or up-regulation IDO1 in MGC-803 and HGC-27, we examined their expression levels by qRT-PCR. As shown in Fig. 3b, the expression of *LOXL2*, *COL6A1*, *COL6A2* and *COL12A1* was obviously attenuated after IDO1 down-regulation in AGS and SGC-7901, while the expression of *LOXL2*, *COL6A1*, *COL6A2* and *COL12A1* was increased after IDO1 overexpression in MGC-803 and HGC-27 (Fig. 3c, all P < 0.01). However, for genes *COL5A1*, *COL8A1* and *COL13A1*, the change of expression levels was not apparent. The obviously regulatory effect of IDO1 on LOXL2, COL6A1, COL6A2 and COL12A1 was also observed at protein levels by Western blot (Fig. 3d).

### COL12A1 is identified as a key cancer-promoting gene and in turn regulates IDO1 expression

**Fig. 4:**
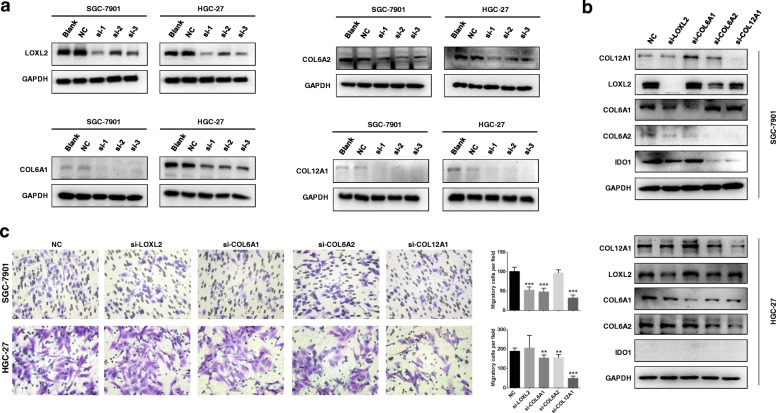
*COL12A1* is identified as a critical cancer-promoting gene enhancing migratory capacity of GC cell. **a** Western blot was utilized to detect expression of LOXL2, CLO6A1, COL6A2 and COL12A1 after transfecting specific siRNAs in SGC-7901 and HGC-27. **b** COL12A1, LOXL2, COL6A1 and COL6A2 were silenced by IDO1, COL12A1, LOXL2, COL6A1 and COL6A2 siRNAs transfection. Three days later, IDO1, COL12A1, LOXL2, COL6A1 and COL6A2 proteins were detected by Western blot. **c** Effects of knockdown of LOXL2, COL6A1, COL6A2 and COL12A1 on SGC-7901 and HGC-27 migratory capacity. “*” represented comparing with “NC” group. **P < 0.01, ***P < 0.001

We used siRNAs to decrease the expression of LOXL2, COL6A1, COL6A2, and COL12A1, and then detected expression level of IDO1 by Western blot. The knockdown effects of siRNAs targeting LOXL2, COL6A1, COL6A2 and COL12A1 were screened, and the most efficient sequences were selected for subsequent experiments (LOXL2: si-1; COL6A1: si-1; COL6A2: si-2; COL12A1: si-3. Fig. 4a). As shown in Fig. 4b, the results indicated that there was a reciprocal positive regulation among the five genes more or less. It was worth noticing that COL12A1 and COL6A2 could positively regulate IDO1. Furthermore, knockdown of COL12A1 significantly suppressed cell migration (Fig. 4c, all *P* < 0.05), although knockdown of LOXL2, COL6A1 and COL6A2 also attenuated cell motility to some extent.

### Both IDO1 and COL12A1 activate MAPK pathway in GC cells

**Fig. 5:**
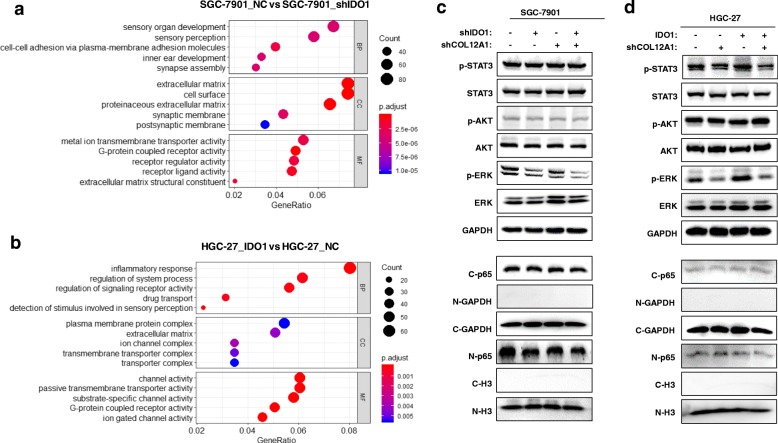
IDO1 and COL12A1 are positively associated with MAPK pathway activation in GC cells. **a** and **b** GO analysis was performed using genes with |log2 (fold change)| > 1 by comparing SGC-7901_NC with SGC-7901_shIDO1, or by comparing HGC-27_IDO1 with HGC-27_NC. Five most involved GO terms were displayed. MF: molecular function, molecular activities of gene products. CC: cellular component, gene products are active. BP: biological process, pathways and larger processes made up of the activities of multiple gene products. **c** Western blot was used to examine p-STAT3, p-AKT, pERK, nucleus and cytoplasm p65 after stable knockdown of IDO1 or/and COL12A1 in SGC-7901. **d** By establishing NC, IDO1, shCOL12A1 and IDO1 + shCOL12A1 stable HGC-27 cell lines, Western blot was used to detect p-STAT3, p-AKT, p-ERK, nucleus and cytoplasm p65. “C” and “N” represented cytoplasm and nucleus protein respectively

To further explore the mechanisms of IDO1-induced GC migration, we constructed IDO1-shRNA lentivirus and IDO1 overexpressing lentivirus and transfected SGC-7901 and HGC-27 cell lines. Then, we successfully constructed SGC-7901_NC, SGC-7901_shIDO1, HGC-27_NC and HGC-27_IDO1 cell lines. After RNA sequencing of these cell lines, we used the genes with |log2 (fold change)| > 1 for GO analysis. The results showed that IDO1 was closely related to extracellular matrix (mainly collagen) synthesis (Fig. 5a, SGC-7901_NC vs SGC-7901_shIDO1; Fig. 5b, HGC-27_IDO1 vs HGC-27_NC. All *P* < 0.001, P. adjust< 0.001), which was consistent with the results of WGCNA analysis. We also found that IDO1 was positively associated with activation of G protein-coupled receptor signaling pathway (all P < 0.001, P.adjust< 0.001). The downstream signaling pathway of G protein-coupled receptor include JAK/STAT3, PI3K/AKT, MAPK and NF-κB [21–24]. We found that double knockdown of IDO1 and COL12A1 could inhibit ERK phosphorylation more effectively than single knockdown of IDO1 or COL12A1 in SGC-7901 (Fig. 5c), while IDO1-mediated phosphorylation of ERK could be reversed by silencing COL12A1 in HGC-27 (Fig. 5d). However, neither IDO1 nor COL12A1 could apparently regulate JAK/STAT3, PI3K/AKT and NF-κB pathways.

### IDO1 and COL12A1 reciprocally induce cell migration via MAPK pathway

**Fig. 6:**
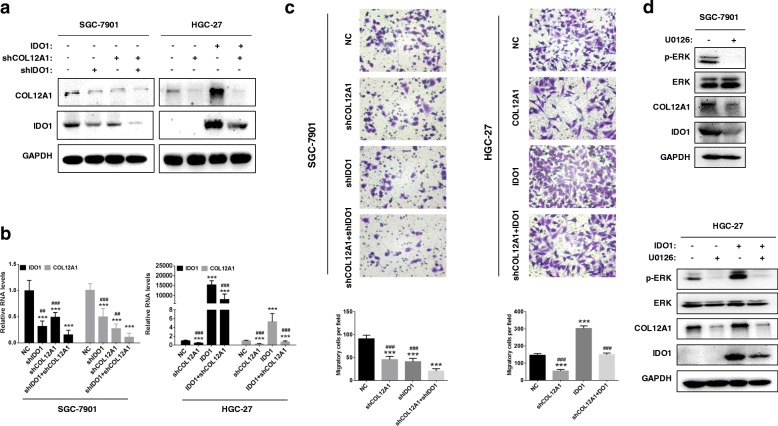
A reciprocal positive regulation between IDO1 and COL12A1 via MAPK to promote GC cell migration. Western blot (**a**) and qRT-PCR (**b**) were used to examine IDO1 and COL12A1 expression in stable knockdown of IDO1 or/and COL12A1 SGC-7901 cell lines, or in HGC-27_NC, HGC-27_IDO1, HGC-27_shCOL12A1 and HGC-27_IDO1 + shCOL12A1 cell lines. **c** Transwell assay was performed to assess the migratory ability of SGC-7901_NC, SGC-7901_shIDO1, SGC-7901_shCOL12A1 and SGC-7901_shIDO1 + shCOL12A1. Similar assay was performed in HGC-27_NC, HGC-27_shCOL12A1, HGC-27_IDO1 and HGC-27_IDO1_shCOL12A1. 200×. **d** Western blot was utilized to detect IDO1 and COL12A1 protein levels after SGC-7901 or HGC-27_IDO1 cells treated with U0126 for 48 h. “*” represented comparing with “NC” group. **P < 0.01, ***P < 0.001. “#” represented comparing with “shIDO1 + shCOL12A1” or “IDO1” group. ^##^*P* < 0.01, ^###^*P* < 0.001

Here, we knocked down both IDO1 and COL12A1 in SGC-7901 cells and found that double knockdown of IDO1 and COL12A1 more effectively decreased expression levels of IDO1 and COL12A1 than single knockdown of IDO1 or COL12A1 (Fig. 6a). In turn, up-regulation of IDO1 in HGC-27 cells could be attenuated by COL12A1 knockdown (Fig. 6a). This phenomenon was verified at the transcriptional level by qRT-PCR (Fig. 6b, all *P* < 0.01). The migration ability of SGC-7901 cells was apparently inhibited by double knockdown of IDO1 and COL12A1, compared to that in single knockdown of IDO1 or COL12A1. In HGC-27 cells, IDO1-enhanced migration ability could be attenuated by COL12A1 knockdown (Fig. 6c, all P < 0.001). After MAPK pathway inhibitor U0126 (1 μM, S1102, SELLECK, Houston, USA) treatment for 48 h, IDO1 and COL12A1 expression was effectively inhibited in SGC-7901 cells. In HGC-27_IDO1 cells, up-regulation of IDO1 and COL12A1 could also be attenuated by U0126 treatment (Fig. 6d).

### Both IDO1-kynurenine and COL12A1-integrin β1 activate MAPK pathway

**Fig. 7:**
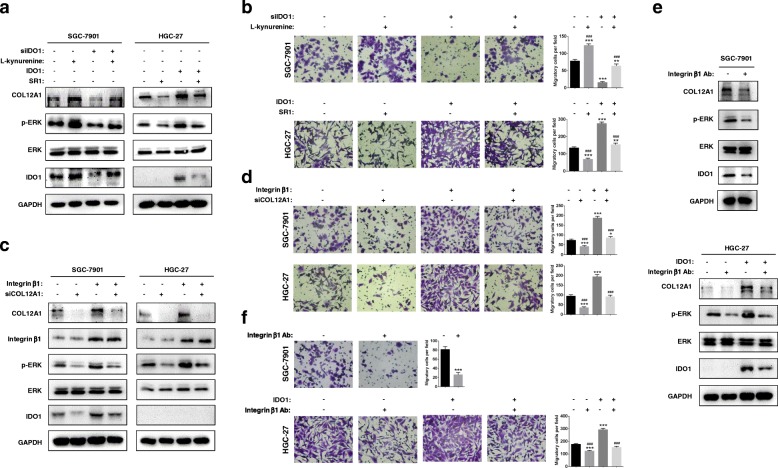
IDO1 metabolite kynurenine and integrin β1 mediate the positive feedback of IDO1-MAPK-COL12A1. **a** After knockdown of IDO1 by siRNA for 48 h, SGC-7901 cells were treated by 10 μM L-kynurenine. After forcing expression of IDO1 by overexpressing eukaryotic plasmid for 48 h, HGC-27 cells were treated by 1 μM SR1. Forty eight hours later, Western blot was used to examine expression of IDO1, COL12A1 and phosphorylated ERK. **b** After knockdown of IDO1 by siRNA or up-regulation of IDO1 by overexpressing eukaryotic plasmid for 48 h, SGC-7901 and HGC-27 cells were treated by 10 μM L-kynurenine or 10 μM SR1 respectively. Forty eight hours later, transwell assay was performed to evaluate migration capacity of GC cells. “*” represented comparing with Con group, and “#” represented comparing with siIDO1 or IDO1 group. **c**-**d** COL12A1 was knocked down by siRNA for 48 h, and then integrin β1 was upregulated by overexpressing eukaryotic plasmid for 48 h in SGC-7901 and HGC-27. Western blot was performed to detect IDO1, COL12A1 and phosphorylated ERK, and transwell assay was conducted to evaluate migration capacity of GC cells. “*” represented comparing with Con group, and “#” represented comparing with integrin β1 group. **e**-**f** SGC-7901 was treated by integrin β1 antibody (3 μg/ml) for 48 h. After IDO1 upregulated by overexpressing eukaryotic plasmid for 48 h, HGC-27 was treated by integrin β1 antibody (3 μg/ml) for 48 h. Expression of IDO1, COL12A1 and phosphorylated ERK was detected by Western blot. GC cell migration ability was evaluated by transwell assay. “*” represented comparing with control group, and “#” represented comparing with IDO1 group. *P < 0.05, **P < 0.01, ***P < 0.001. ##*P* < 0.01, ###*P* < 0.001

IDO1 enzyme plays a central role in catalyzing tryptophan to kynurenine. We analyzed the effects of IDO1 metabolite L-kynurenine on cancer cells. After incubation of L-kynurenine (1-100 μM) for 48 h, both cell growth and migration ability were promoted, especially at the concentration of 10 μM (Additional file 5: Figure S1 and Additional file 6: Figure S2, all *P* < 0.05). Down-regulation of IDO1 inhibited expression of COL12A1 and phosphorylated ERK, proliferation and migration, which could be reversed by L-kynurenine (10 μM, Fig. 7a, Additional file 7: Figure S3a, all *P* < 0.05). L-kynurenine is an agonist for aryl hydrocarbon receptor (AhR), which can be antagonized by StemRegenin 1 (SR1). After incubation with SR1 for 48 h, the expression of COL12A1 and phosphorylated ERK induced by IDO1 overexpression was decreased in HGC-27 cells, and the migration ability was also inhibited along with the SR1 incubation (1 μM, Fig. 7a, Fig. 7b, and Additional file 3: Figure S3b, all *P* < 0.05). Type XII collagen (encoded by *COL12A1*), a member of the FACIT (fibril-associated collagens with interrupted triple helices) collagen family, mediates interactions between the fibrils and the surrounding matrix. COL12A1 may activate several intracellular signaling pathways via collagen I/integrin β1 [25, 26]. Therefore, we enforced integrin β1 expression after knocking down COL12A1 in SGC-7901 and HGC-27. Down-regulation of COL12A1, IDO1 and phosphorylated ERK as well as decreased migration ability were observed by knockdown of COL12A1, which were partly restored by integrin β1 overexpression (Fig. 7c and Fig. 7d, all *P* < 0.05). We further blocked integrin β1 by using integrin β1 monoclonal antibody (3 μg/ml). In SGC-7901 cells, blockage of integrin β1 could inhibit cell migratory ability and expression of IDO1, COL12A1 and phosphorylated ERK (Fig. 7e and Fig. 7f, all *P* < 0.05). In HGC-27 cells, up-regulation of IDO1 enhanced cell migratory ability and increased expression levels of IDO1, COL12A1 and phosphorylated ERK, which could be reversed by integrin β1 blockage (Fig. 7e and f, all *P* < 0.05).

### Identification of interdependence between IDO1 and COL12A1 on promoting metastasis

**Fig. 8:**
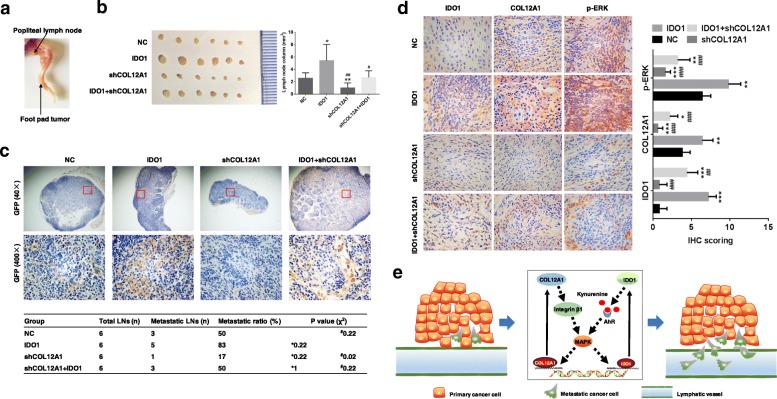
IDO1 and COL12A1 synergistically promote GC metastasis. **a** Cancer cells were inoculated in foot-pad of nude mice to establish xenografted tumor metastasis model. **b** After inoculation with HGC-27_NC, HGC-27_shCOL12A1, HGC-27_IDO1 and HGC-27_IDO1_shCOL12A1 in nude mice foot-pad for 3 weeks, popliteal lymph nodes (left) were removed and their volumes (right) were measured. **c** IHC staining of GFP was performed to assess metastatic lymph node ratio in different groups (χ^2^ test). **d** IHC staining of IDO1, COL12A1 and p-ERK in primary foot pad tumors. **e** A positive feedback between IDO1 and COL12A1 to promote GC metastasis. Both IDO1-kynurenine and COL12A1-integrin β1 could activate MAPK pathway, which promoted IDO1 and COL12A1 transcriptional activation. The feedback would powerfully enhance GC cell metastasis. “*” represented comparing with “NC” group. *P < 0.05, **P < 0.01, ***P < 0.001. “#” represented comparing with “IDO1” group. ^#^*P* < 0.05, ^##^*P* < 0.01, ^###^*P* < 0.001

To investigate the effects of IDO1 and COL12A1 on GC metastasis in vivo, we injected HGC-27_NC, HGC-27_IDO1, HGC-27_shCOL12A1 and HGC-27_IDO1 + shCOL12A1 cells into mouse footpads to construct a footpad xenograft model (Fig. 8a). Three weeks later, the mice were sacrificed, and popliteal lymph nodes were removed and node sizes were measured. The lymph nodes in HGC-27_IDO1 group revealed the largest size, while the lymph nodes in HGC-27_shCOL12A1 group disclosed the smallest size (Fig. 8b, all *P* < 0.05). Given that the GFP was labeled in lentivirus plasmid, the transfected cancer cells could be marked by GFP. Therefore, we could regard a lymph node as a metastatic one where cancer cells were identified by IHC staining of GFP (Fig. 8c). Accordingly, we calculated the metastatic rate of lymph nodes in different groups. Among the four groups, the metastatic rate of lymph nodes was the highest (83%) in HGC-27_IDO1 group, followed by HGC-27_NC group (50%), HGC-27_IDO1 + shCOL12A1 group (50%) and HGC-27_shCOL12A1 group (17%) (Fig. 8c). The metastatic rate of lymph nodes was significantly different between HGC-27_IDO1 group and HGC-27_shCOL12A1 group by chi-square test (*P* < 0.05, Fig. 8c). By IHC staining, we examined IDO1, COL12A1 and phosphorylation ERK expression in primary tumor of the four groups. Their expression was the strongest in HGC-27_IDO1, and the weakest in the HGC-27_shCOL12A1 group (Fig. 8d, all *P* < 0.05). Our experiments well clarified a reciprocal positive regulation between IDO1 and COL12A1 to promote tumor metastasis, which was mediated by IDO1 metabolite kynurenine and integrin β1 (Fig. 8e).

## Discussion

Increased IDO1 of cancer cells directly resulted in tryptophan exhaustion in microenvironment and reduced the activation of immune cells [5]. By GSEA analysis, IDO1 was significantly associated with pathways involved in immune activation, including T cell and NK cell. Therefore, despite the apparent immune cell infiltration, an immune tolerance was induced [27, 28]. Expression of IDO1 played an important role on tumor migration and invasion in melanoma, bladder cancer and lung cancer [6–9]. IDO1 could effectively promote the migration of GC cell in our study. In addition, IDO1 revealed a pro-proliferative effect on melanoma cells [29]. To clarify the mechanisms of IDO1 on promoting migration, WGCNA analysis was performed to unravel most closely IDO1-correlated module, which was involved in extracellular matrix (mainly including collagens). Poormasjedi-Meibod et al. found that the expression of type I collagen could be induced by kynurenine, a tryptophan metabolite via AhR in skin fibroblasts [30]. We also found that IDO1 was positively correlated with extracellular matrix by GO analysis. Expression of collagens in tumor tissues could ignite tumor invasion and metastasis via various pathways. Vaniotis et al. found that type IV collagen could induce liver metastasis of lung cancer by increasing CCL5 and CCL7 synthesis [31]. Collagen IV could also activate DDR1 and AKT to promote invasion and migration of myeloid leukemia cells [32]. Epithelial mesenchymal transition was a critical mechanism of cancer cell motility induced by collagens [33]. TGF-β is a classical growth factor that promotes tumor metastasis. Cheon et al. found that TGF-β-induced migration and invasion of ovarian serous cystadenocarcinoma depended on the expression of multiple collagen-remodeling genes, such as *COL11A1*, *COL5A1* and *COL6A2* [34]. In our study, IDO1 metabolites enhance cell motility by increasing extracellular matrix expression, especially COL12A1 gene. Fei et al. found that extracellular matrix around cancer cells could construct a barrier which could prevent T cells from entering tumor tissue and killing cancer cells [35, 36]. Our results also support that IDO1-mediated immunosuppression may be partly due to increased extracellular collagen matrix surrounding cancer cells.

By establishing co-expression network analysis of collagen, five hub genes (*AXL*, *SGCE*, *CLO12A1*, *ANTXR1* and *LOXL2*) were picked up. Meanwhile, we also plotted a correlation coefficient matrix diagram based on hierarchical cluster analysis among IDO1 and collagen genes. IDO1 and 6 collagen genes (*COL5A1*, *COL6A1*, *COL6A2*, *COL8A1*, *COL12A1*, *COL13A1*) showed strong correlations and clustered together. Further validation in vitro revealed IDO1 positively regulated LOXL2, COL6A1, COL6A2 and COL12A1. These genes were found to participate in collagen synthesis and closely associated with cancer cell migration [34, 37–39]. Up-regulation of IDO1, LOXL2 and COL12A1 was also verified in GC tissues from TCGA database. By further in vitro experiments, COL12A1 had been found playing a key role on cell migration. COL12A1, a gene encoding collagen type XII alpha 1 chain, is a typical collagen-organizer molecule involved in collagen cross-linking in cancer microenvironment [40]. This gene can be expressed in fibroblasts and cancer cells [40]. Although previous studies revealed that COL12A1 was closely related to tumor migration, invasion and metastasis in breast and colorectal cancer [39–41], but the underlying mechanisms were still unclear.

Our study firstly clarified that the enhancing migratory ability of cancer cells was interdependent between COL12A1 and IDO1. That is, there is a reciprocal positive regulatory correlation between IDO1 and COL12A1. The positive feedbacks among different genes play important roles on tumor progression. For example, inactivating mTOR by miR-3188 resulted in suppression of p-PI3K/p-AKT/c-JUN pathway, and mTOR in turn enhanced miR-3188 expression in nasopharyngeal carcinoma [19, 42]. GO analysis confirmed that IDO1 was closely associated with the activation of G protein-coupled receptor signaling pathway, which directly activated JAK/STAT3, PI3K/AKT, MAPK and NF-κB pathways [21–24]. We screened above pathways, and identified MAPK pathway as the downstream of IDO1 and COL12A1. IDO1 inhibitor and IDO1 metabolite kynurenine have been reported to be involved in MAPK pathway activation [43]. In cutaneous squamous cell carcinoma, Type VII collagen could induce cancer cell invasion and migration by activating MAPK pathway [44]. Meanwhile, MAPK activation could increase collagen expression in fibroblast [45, 46].

IDO1 is an enzyme which regulates concentration of an amino acid. Li et al. proposed that IDO1 metabolite kynurenine could activate MAPK pathway in dermal fibroblast [43]. Our results firstly indicated that IDO1 activated MAPK pathway via IDO1 metabolite kynurenine/AhR pathway in GC. COL12A1 encodes a member of the fibril-associated collagens with interrupted triple helices (FACITs), which construct a homotrimer with collagen I and collagen III, and mediate interactions between fibrils and surrounding matrix [47, 48]. Collagen I, as a ligand of integrin β1, could activate downstream pathways of integrin β1 [26]. Mohammed-Amine found that collagen I/integrin β1 could increase expression of ABCC1/MRP-1 transporter by activating MAPK pathway [25]. In this study, we found that COL12A1 promoted phosphorylation of ERK via integrin β1 pathway. Therefore, we speculated that collagen XII stabilized the fibril spacing, and mediated collagen fibrils interactions with integrin β1, which resulted in ERK phosphorylation in GC cells.

## Conclusions

We report a novel function and mechanism of IDO1-mediated GC progression. IDO1 is positively related to the module of extracellular matrix and collagen processing. Of those related genes, COL12A1 is identified as the critical gene on promoting cancer migration. IDO1 metabolite kynurenine and COL12A1/integrin β1 constitute a reciprocal positive feedback via MAPK signaling pathway, which contributes to GC metastasis. Our results evidently suggest a useful therapeutic approach for targeting IDO1 and COL12A1.

## Additional files

Additional file 1: **Table S1.** SiRNA sequences used in this study. (DOCX 12 kb)

Additional file 2: **Table S2.** Primer sequences used in this study. (DOCX 13 kb)

Additional file 3: **Table S3.** Antibodies used in this study. (DOCX 16 kb)

Additional file 4: Supplementary data. (XLSX 1337 kb)

Additional file 5: **Figure S1.** IDO1 metabolite kynurenine promotes GC cell proliferation. L-kynurenine (0-100 μM) was used to treat GC cells at different concentrations for 48 h, and CCK8 assay was performed to assess GC cell proliferation ability. **P < 0.01, ***P < 0.001. (PDF 42 kb)

Additional file 6: **Figure S2.** IDO1 metabolite kynurenine promotes GC cell migration. SGC-7901 and HGC-27 cells were treated with different concentrations of L-kynurenine (0-100 μM), and transwell assay was performed to assess migration ability. *P < 0.05, **P < 0.01, ***P < 0.001. (PDF 397 kb)

Additional file 7: **Figure S3.** Kynurenine mediates IDO1-induced GC cell proliferation. a) After knockdown of IDO1 by siRNA for 48 h, GC cells were treated by 10 μM L-kynurenine. CCK8 assay was performed to evaluate proliferation capacity of GC cells. b) After enforcing expression of IDO1 by overexpressing eukaryotic plasmid for 48 h, GC cells were treated by 1 μM SR1. CCK8 assay was conducted to assess GC cell proliferation ability. “*” represented comparing with Con group, and “#” represented comparing with siIDO1 or IDO1 group. *P < 0.05, ***P < 0.001. ##*P* < 0.01, ###*P* < 0.001. (PDF 21 kb)

## Data Availability

All data generated or analyzed during this study are included in this published article and its Additional files.

## Abbreviations

CCLE: Cancer Cell Line Encyclopedia
GC: gastric cancer
GO: Gene Ontology
GSEA: Gene Set Enrichment Analysis
IDO1: Indoleamine 2,3-dioxygenase 1
WGCNA: Weighted Gene Co-expression Network Analysis
