# Supplementary material for: A positive feedback between IDO1 metabolite and COL12A1 via MAPK pathway to promote gastric cancer metastasis
Source: J Exp Clin Cancer Res. 2019 Jul 17;38:314. doi: 10.1186/s13046-019-1318-5 (PMC6637527; doi:10.1186/s13046-019-1318-5)
Supplement: Supplementary file 1 — Table S1. SiRNA sequences used in this study. (DOCX 12 kb) [file 13046_2019_1318_MOESM1_ESM.docx]

Table S1. SiRNA sequences used in this study.

| Gene (siRNA secquence) | Sense（5'-3'） | Antisense（5'-3'） |
| --- | --- | --- |
| IDO1 | | |
| si-1 | GCAGACUGUGUCUUGGCAATT | UUGCCAAGACACAGUCUGCTT |
| si-2 | GGGAAGACCCAAAGGAGUUTT | AACUCCUUUGGGUCUUCCCTT |
| si-3 | GGAGAAUAAGACCUCUGAATT | UUCAGAGGUCUUAUUCUCCTT |
| COL6A2 | | |
| si-1 | GGGCCUCCUUCAUCAAGAATT | UUCUUGAUGAAGGAGGCCCTT |
| si-2 | GCAGGCCUGGAUUCAGCUATT | UAGCUGAAUCCAGGCCUGCTT |
| si-3 | GGCUGGGUGCCAUCGCUAATT | UUAGCGAUGGCACCCAGCCTT |
| LOXL2 | | |
| si-1 | GCAACCGGCUCCUGAGUAUTT | AUACUCAGGAGCCGGUUGCTT |
| si-2 | CCGGGUGGAGGUGUACUAUTT | AUAGUACACCUCCACCCGGTT |
| si-3 | CCAGAUAGAGAACCUGAAUTT | AUUCAGGUUCUCUAUCUGGTT |
| COL12A1 | | |
| si-1 | GCAAUAAACACCUUCCCUUTT | AAGGGAAGGUGUUUAUUGCTT |
| si-2 | GGAGAACACUGGAGAACUUTT | AAGUUCUCCAGUGUUCUCCTT |
| si-3 | GCUGAUGAAGUCGAAUUAATT | UUAAUUCGACUUCAUCAGCTT |
| COL6A1 | | |
| si-1 | CCAAGCGCUUCAUCGACAATT | UUGUCGAUGAAGCGCUUGGTT |
| si-2 | GCCAGACCAUCGACACCAUTT | AUGGUGUCGAUGGUCUGGCTT |
| si-3 | GCAUUGGCCUGCAGAACUUTT | AAGUUCUGCAGGCCAAUGCTT |
