# Supplementary material for: A positive feedback between IDO1 metabolite and COL12A1 via MAPK pathway to promote gastric cancer metastasis
Source: J Exp Clin Cancer Res. 2019 Jul 17;38:314. doi: 10.1186/s13046-019-1318-5 (PMC6637527; doi:10.1186/s13046-019-1318-5)
Supplement: Supplementary file 2 — Table S2. Primer sequences used in this study. (DOCX 13 kb) [file 13046_2019_1318_MOESM2_ESM.docx]

Table S2. Primer sequences used in this study.

| Gene | Forward | Reverse |
| --- | --- | --- |
| IDO1 | GATCATCTCACAGACCACAAGTCACAG | CTTGGAGAGTTGGCAGTAAGGAACAG |
| COL12A1 | TATTGTGTTCTTGACTGATGCCTCCTG | AGACTTGACCTCATCGCTGTATTGC |
| COL6A1 | GAGCTGGTCAAGTTCGAGCC | CCACTGCAGGCTCTTGATGG |
| COL6A2 | TGCTCCGTGCTCCTGCTCTG | ATGGTGACGCTCTCCGAGGTG |
| LOXL2 | ACGGAGGATGTCGGTGTGGTG | GCTTGCGGTAGGTTGAGAGGATG |
| COL5A1 | CGACATGCGTCTTCCCTGAC | GAGCAGTTTCCCACGCTTGA |
| COL8A1 | AGCTCACACGTTCACCAACTCAC | AGATGCTGCTCTGCCTTCACAAC |
| COL13A1 | GCTGCTGCCTCTCCTCAATTCAG | TGGATGCTGGCCTGGCTCTG |
