# Supplementary material for: A positive feedback between IDO1 metabolite and COL12A1 via MAPK pathway to promote gastric cancer metastasis
Source: J Exp Clin Cancer Res. 2019 Jul 17;38:314. doi: 10.1186/s13046-019-1318-5 (PMC6637527; doi:10.1186/s13046-019-1318-5)
Supplement: Supplementary file 3 — Table S3. Antibodies used in this study. (DOCX 16 kb) [file 13046_2019_1318_MOESM3_ESM.docx]

Table S3. Antibodies used in this study.

| Antibody | Company | Application (density) |
| --- | --- | --- |
| Rabbit IDO1 (D5J4E™) monoclonal antibody | CST (86630) , Boston, USA | Western blot (1:1000), IHC (1:50) |
| Rabbit STAT3 (D3Z2G) monoclonal antibody | CST (12640S) , Boston, USA | Western blot (1:1000) |
| Rabbit Phospho-Stat3 (Tyr705) (D3A7) monoclonal antibody | CST (9145S) , Boston, USA | Western blot (1:1000) |
| Rabbit Phospho-p44/42 MAPK (Erk1/2) (Thr202/Tyr204) (D13.14.4E) monoclonal antibody | CST (4370S) , Boston, USA | Western blot (1:1000) |
| Rabbit p44/42 MAPK (Erk1/2) (137F5) monoclonal antibody | CST (4695S) , Boston, USA | Western blot (1:1000) |
| Rabbit Akt (pan) (C67E7) monoclonal antibody | CST (4691S) , Boston, USA | Western blot (1:1000) |
| Rabbit Phospho-Akt (Ser473) (D9E) monoclonal antibody | CST (4060S) , Boston, USA | Western blot (1:1000) |
| Rabbit NF-κB p65 (D14E12) monoclonal antibody | CST (8242S) , Boston, USA | Western blot (1:1000) |
| Rabbit COL6A1 polyclonal antibody | Abcam (ab151422), Cambridge, UK | Western blot (1:1000) |
| Rabbit COL6A2 monoclonal antibody | Abcam (ab180855), Cambridge, UK | Western blot (1:1000) |
| Rabbit LOXL2 polyclonal antibody | Abcam (ab96233), Cambridge, UK | Western blot (1:1000) |
| Rabbit COL12A1 polyclonal antibody | Abcam (ab121304), Cambridge, UK | Western blot (1:1000), IHC (1:50) |
| Rabbit Integrin beta 1 monoclonal antibody [EPR1040Y] | Abcam (ab134179), Cambridge, UK | Western blot (1:500) |
| Human Integrin beta 1/CD29 monoclonal antibody | R&D (MAB17781), Minnesota, USA | Blockade of Receptor-ligand Interaction (3μg/ml) |
| Rabbit Histone H3 polyclonal antibody | Abcam (ab1791), Cambridge, UK | Western blot (1:5000) |
| Rabbit GFP polyclonal antibody | proteintech (50430-2-AP), Wuhan, China | IHC (1:50) |
| HRP-Conjugated GAPDH Antibody | proteintech (HRP-60004), Wuhan, China | WB (1:3000) |
