# Supplementary figures and images for: A positive feedback between IDO1 metabolite and COL12A1 via MAPK pathway to promote gastric cancer metastasis
Source: J Exp Clin Cancer Res. 2019 Jul 17;38:314. doi: 10.1186/s13046-019-1318-5 (PMC6637527; doi:10.1186/s13046-019-1318-5)

# SGC-7901

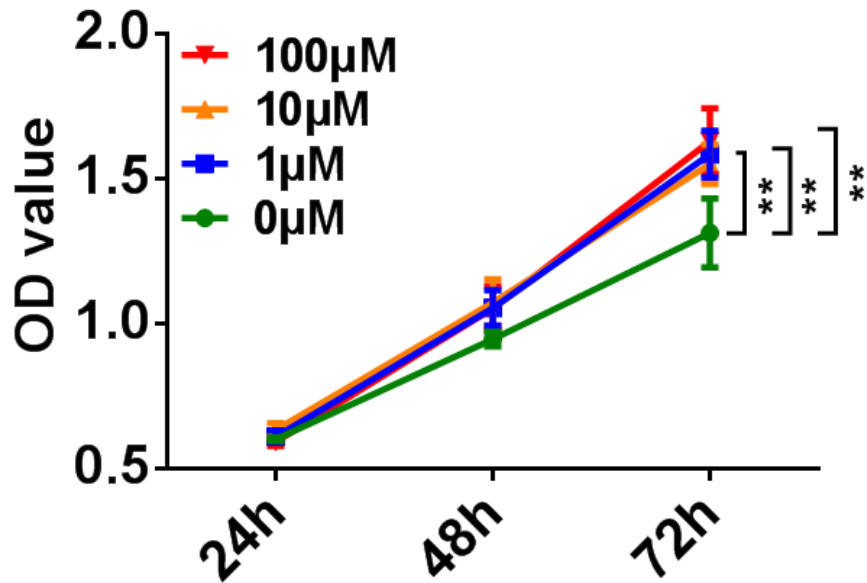

# HGC-27

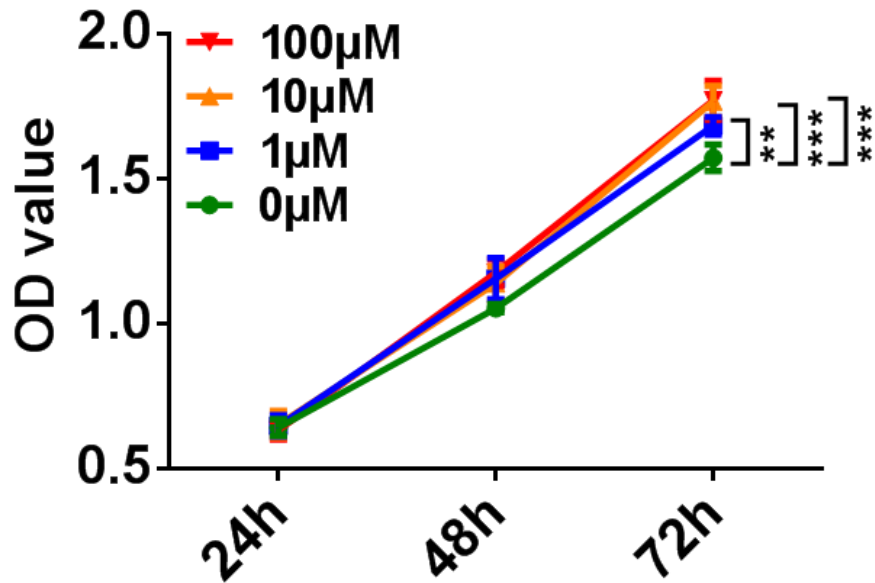

Supplement: Supplementary file 5 — Figure S1. IDO1 metabolite kynurenine promotes GC cell proliferation. L-kynurenine (0-100 μM) was used to treat GC cells at different concentrations for 48 h, and CCK8 assay was performed to assess GC cell proliferation ability. **P < 0.01, ***P < 0.001. (PDF 42 kb) [file 13046_2019_1318_MOESM5_ESM.pdf]

SGC-7901

0 $\mu$ M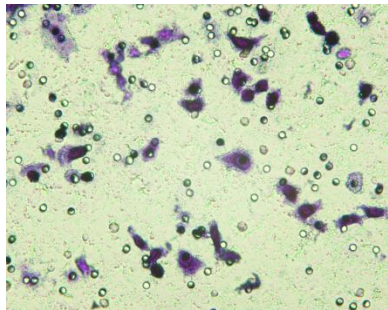1 $\mu$ M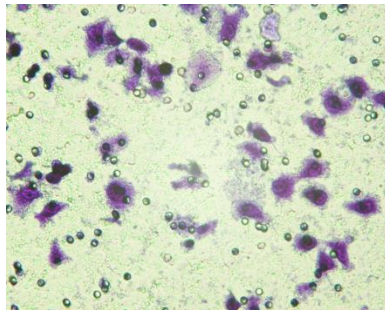10 $\mu$ M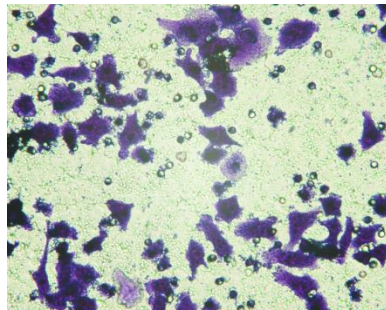100 $\mu$ M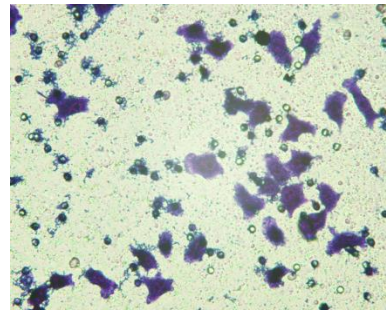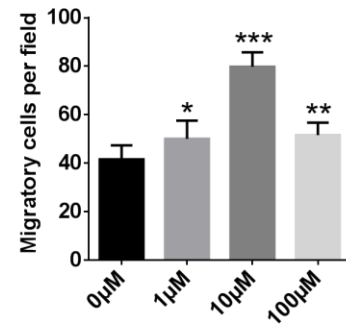

HGC-27

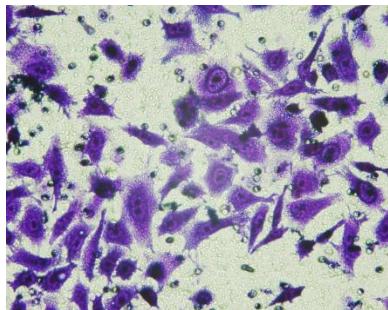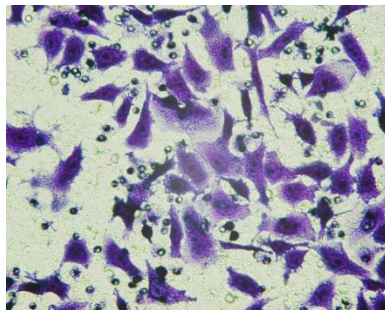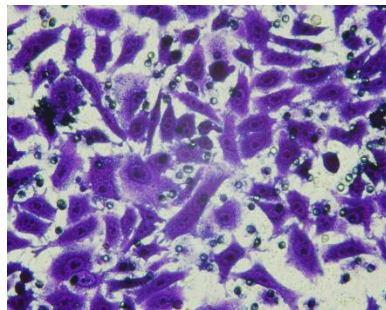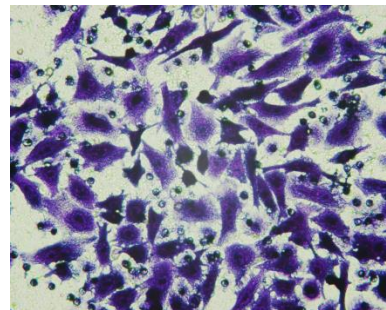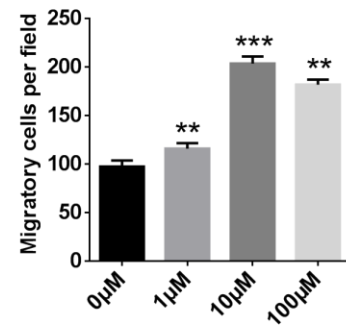

Supplement: Supplementary file 6 — Figure S2. IDO1 metabolite kynurenine promotes GC cell migration. SGC-7901 and HGC-27 cells were treated with different concentrations of L-kynurenine (0-100 μM), and transwell assay was performed to assess migration ability. *P < 0.05, **P < 0.01, ***P < 0.001. (PDF 397 kb) [file 13046_2019_1318_MOESM6_ESM.pdf]

**a****SGC-7901**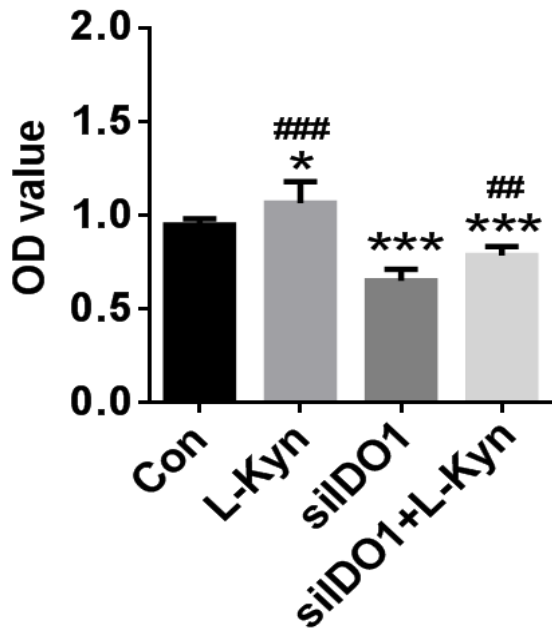**b****HGC-27**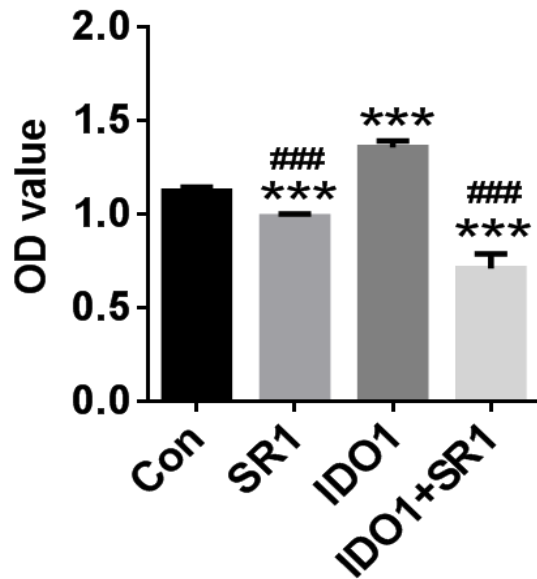

Supplement: Supplementary file 7 — Figure S3. Kynurenine mediates IDO1-induced GC cell proliferation. a) After knockdown of IDO1 by siRNA for 48 h, GC cells were treated by 10 μM L-kynurenine. CCK8 assay was performed to evaluate proliferation capacity of GC cells. b) After enforcing expression of IDO1 by overexpressing eukaryotic plasmid for 48 h, GC cells were treated by 1 μM SR1. CCK8 assay was conducted to assess GC cell proliferation ability. “*” represented comparing with Con group, and “#” represented comparing with siIDO1 or IDO1 group. *P < 0.05, ***P < 0.001. ##P < 0.01, ###P < 0.001. (PDF 21 kb) [file 13046_2019_1318_MOESM7_ESM.pdf]
